# Safety and efficacy of continuous intra-arterial infusion of heparin administration in mechanical thrombectomy for acute ischemic stroke: a single-center retrospective study

**DOI:** 10.3389/fneur.2025.1736654

**Published:** 2026-01-16

**Authors:** Tao Meng, Xuefei Wang, Liu Yang, Jinping Wang, Pei Wang, Hui Wang, Yueqin Zhou, Oumei Cheng

**Affiliations:** 1 Department of Neurology, The First Affiliated Hospital of Chongqing Medical University, Chongqing, China; 2 Department of Neurology, Chongqing University Central Hospital, Chongqing, China; 3 Chongqing Key Laboratory of Emergency Medicine, Chongqing, China; 4 Key Laboratory of Major Brain Disease and Aging Research (Ministry of Education), Chongqing Medical University, Chongqing, China

**Keywords:** heparin, infusion, intra-arterial, stroke, thrombectomy

## Abstract

**Background and purpose:**

The use of heparin during mechanical thrombectomy (MT) for acute large vessel occlusion ischemic stroke (LVO-AIS) is controversial, with no unified standard on its administration methods and efficacy. This study aims to investigate the effectiveness and safety of continuous intra-arterial infusion of heparin administration during MT in real-world practice.

**Materials and methods:**

A single-center retrospective study included consecutive LVO stroke patients treated with mechanical thrombectomy at Chongqing University Central Hospital (August 2022–January 2024). Participants were stratified by intraprocedural heparin administration: (1) arterial heparin Group-continuous intra-arterial heparinization via high-pressure infusion (Heparin 1,000 IU was diluted in 500 ml of 0.9% sodium chloride solution and connected to both the guiding catheter and the intermediate catheter. The infusion bag was replaced as needed according to the duration of the procedure) at a conventional drip rate; (2) non-additional heparin Group-standard heparin solution flushing withoutusing additional anticoagulant. The main outcome were 3-months functional independence, defined as a modified Rankin Scale (mRS) ≤ 2. The main Safety outcome were defined as symptomatic intracranial hemorrhage (sICH) in 24 h.

**Results:**

A total of 98 patients were eligible for analysis: 54 in the Arterial heparin Group and 44 in the Non-additional heparin Group. Continuous intra-arterial infusion of heparin administration during MT had a higher rates of functional independence (57.4 vs. 36.4%, adjusted *P* = 0.035), no significant impact on recanalization rate, sICH, distal embolization, or mortality (adjusted *P* > 0.05). However, the admission NIHSS score [odds ratio 1.225 (1.096–1.370), *P* < 0.01] was identified as independent predictor of unfavorable outcomes, while anticoagulant therapy during hospitalization [odds ratio 0.209 (0.067–0.653), *P* < 0.01] was a protective factor. Aspiration was a protective factor against sICH [odds ratio 0.009 (0.00–0.845), *P* = 0.042].

**Conclusion:**

Our study suggests that continuous intra-arterial infusion of heparin administration during mechanical thrombectomy for acute large vessel occlusion ischemic stroke may be safe and is associated with higher rates of favorable outcomes. Further prospective research is needed to validate these findings.

## Introduction

Mechanical Thrombectomy (MT) has become the primary treatment for acute large vessel occlusion ischemic stroke (AIS-LVO) (1). Heparin, a commonly used anticoagulant, is primarily utilized in MT to prevent thrombus reformation and distal embolic events (2). However, in real-world MT practice, the use and dosage of heparin largely depend on the personal decisions of neurointerventionalists, leading to variations between different medical centers and a lack of standardized protocols (3, 4). Global practice patterns for periprocedural heparin use during endovascular stroke treatment vary widely, with the majority of interventionalists administering heparin via flush fluids and highly variable dosing, reflecting the absence of international consensus on optimal anticoagulation strategies (5). The main concern is that indiscriminate heparin administration may elevate the risk of hemorrhagic events. Consequently, there is an urgent need to identify a safe, effective, and practical strategy for heparin use during MT. At our stroke center, some neurointerventionalists have adopted a strategy of continuous intra-arterial heparin administration via high-pressure infusion throughout the MT procedure. Based on this institutional practice, we conducted a retrospective analysis of clinical data from patients with AIS-LVO who underwent MT at the Advanced Stroke Center of Chongqing University Central Hospital between August 2022 and January 2024. The aim of this study was to investigate the efficacy and safety of continuous intra-arterial infusion of heparin administration during MT.

## Materials and methods

### Inclusion and exclusion criteria

A retrospective analysis was conducted on 98 patients from advanced stroke center of Chongqing University Central Hospital who underwent MT for AIS-LVO between August 2022 and January 2024. The inclusion criteria were: (1) age ≥18 years; (2) pre-stroke modified Rankin Scale (mRS) score ≤ 2; (3) stroke onset within 6 h for both anterior and posterior circulation strokes, (4) proximal large vessel occlusions, including the internal carotid artery, middle cerebral artery M1/M2 segments, basilar artery; (5) patients within the extended time window (6–24 h) screened according to DAWN and DEFUSE 3 criteria; and (6) patients eligible for intravenous thrombolysis (IVT) according to guidelines received a thrombolysis-bridged MT, those with contraindications or who refused IVT underwent direct MT.

Exclusion criteria included: (1) initial National Institutes of Health Stroke Scale (NIHSS) score < 5; and (2) lossing to follow-up.

### Baseline data collection

The following data were collected for analysis: (1) demographic characteristics: age, gender, and marital status; (2) medical history: history of hypertension, diabetes, atrial fibrillation, previous stroke, smoking (defined as smoking for more than 6 months with a daily consumption of more than 10 cigarettes), and alcohol abuse (defined as alcohol consumption for more than 6 months with a daily intake of at least 30 g or weekly intake of 210 g); (3) vascular risk factors: time intervals from onset to puncture (OTP) and from onset to reperfusion (OTR), initial NIHSS score, and ischemic stroke subtypes according to the TOAST.

### MT procedure

According to current guidelines, eligible patients underwent MT after evaluation of indications. The methods included stent retriever thrombectomy, aspiration thrombectomy, or a combination of both. In cases of MT failure, the treating physician decided on rescue therapies, which primarily included intra-arterial/intravenous administration of tirofiban, balloon angioplasty, and stent placement.

### Grouping and treatment measures

This retrospective study was divided into two groups:

(1) Arterial heparin group: in addition to the standard heparin solution for flushing vascular sheaths and catheters, patients received continuous intra-arterial infusion of heparin administration (via high-pressure infusion): 1,000 IU heparin was added to each 500 ml saline solution and connected to both the guiding catheter and the intermediate catheter at conventional intravenous drip rate, replaced according to the duration of the procedure.
(2) Non-additional heparin group: patients received heparin according to standard angiographic practices (heparin solution composed of 12,500 IU heparin in 1,000 ml saline, primarily used for flushing vascular sheaths and catheters) and continuous normal saline via high-pressure infusion, without additional heparin administration.

### Efficacy and safety outcomes

Primary efficacy measure: functional independence at 90 days: defined as a modified Rankin Scale (mRS) score of ≤ 2. Secondary efficacy measures: vessel recanalization: defined as expanded Thrombolysis in Cerebral Infarction score (eTICI) 2b-3 on final angiography during MT. Primary safety measure: symptomatic intracranial hemorrhage (sICH) Within 24 h Post-MT: evaluated by CT or MRI and defined by the European Cooperative Acute Stroke Study III (ECASS-III) criteria as an NIHSS score worsening by ≥4 points related to intracerebral hemorrhage. Mortality rate: defined as an mRS score of 6 at 90 days. Secondary safety measures: any intracerebral hemorrhage (ICH) on Follow-up CT or MRI; distal embolization during MT.

Clinical outcomes, including sICH and 90-day mRS, were assessed using predefined criteria by clinicians not directly involved in the endovascular procedure. Neuroimaging outcomes were independently reviewed by experienced readers blinded to clinical data and treatment allocation. Although assessors were not formally blinded, outcome evaluation followed standardized definitions.

### Statistical analysis

We compared baseline characteristics and all efficacy and safety outcome measures between the heparin group and the non-heparin group, including long-term functional outcomes, mortality rate, vessel recanalization rate, symptomatic intracerebral hemorrhage (sICH), total intracerebral hemorrhage (ICH), and distal embolization. For categorical variables, chi-square tests were used. Continuous variables were first tested for homogeneity of variances and normal distribution; based on these tests, either analysis of variance (ANOVA) or Kruskal–Wallis tests were applied. Additionally, we fitted logistic regression models to explore the impact of heparin on all safety and efficacy outcome measures after adjusting for multiple factors (incorporating these factors as covariates in the model). Moreover, to assess the correlation between heparin and other baseline characteristics with the probability of sICH and adverse outcomes (evaluated based on mRS scores), we performed both univariate and multivariate logistic regression analyses.

All statistical analyses were conducted with SPSS software version 26. Two-tailed *P* < 0.05 were considered as statistically significant.

## Results

### Demographics and baseline characteristics

A total of 98 patients with LVO-AIS who underwent MT were included in the analysis. The baseline characteristics and procedural details are presented in Table 1. Among these patients, 54 (55.1%) were in the arterial-heparin group and 44 (44.8%) were in the non-additional heparin group. The arterial-heparin group had a higher proportion of patients undergoing bridging therapy [43 (79.6%) vs. 27 (61.4%)], post-operative anticoagulation [24 (44.4%) vs. 9 (20.5%)], and direct aspiration [46 (85.2%) vs. 26 (59.1%)]. Conversely, the non-additional heparin group had a higher proportion of smokers [23 (52.3%) vs. 16 (29.6%)] and patients receiving combined aspiration and stent retriever thrombectomy [12 (27.3%) vs. 5 (9.3%)]. There were no significant differences in other baseline characteristics between the two groups (Table 1).

**Table 1:** Baseline characteristics of patients according to heparin used.

**Characteristic**	**All patients**	**Intra-arterial heparin**	**Non-additional heparin**	***P*-value**
	***n*** = **98**	***n*** = **54**	***n*** = **44**	
Age, mean ± SD	68.09 ± 13.75	68.98 ± 13.38	67 ± 14.27	0.481
Male, (*n* %)	56 (57.1)	29 (53.7)	27 (61.4)	0.446
Onset less than 6 h, (*n* %)	84 (85.7)	49 (90.7)	35 (79.6)	0.115
Bridging IVT, (*n* %)	70 (71.4)	43 (79.6)	27 (61.4)	0.046 < 0.05
Admission NIHSS, median (IQR)	18 (15–20)	18 (15–19)	18.5 (14.75–21)	0.236
OTP time, median (IQR), min	111.5 (86.5–147.8)	105 (76.3–137.3)	118.5 (94.8–158.5)	0.059
OTR time, median (IQR), min	50 (40–73.8)	50 (40–65)	47.5 (34.3–76.3)	0.454
Anticoagulation (*n* %)	33 (33.7)	24 (44.4)	9 (20.5)	0.012 < 0.05
Antiplatelet (*n* %)	42 (42.9)	21 (38.9)	21 (47.7)	0.379
**Vascular risk factors (*****N*** **%)**				
Previous ischemic stroke	16 (16.3)	10 (18.5)	6 (13.6)	0.515
Hypertension	48 (49.0)	26 (48.2)	22 (50.0)	0.855
Diabetes mellitus	12 (12.2)	6 (11.1)	6 (13.6)	0.704
Atrial fibrillation	46 (46.9)	23 (42.6)	23 (52.3)	0.34
Moderate to heavy alcohol consumption	29 (29.6)	17 (31.5)	12 (27.3)	0.65
Previous or current smoker	41 (41.8)	18 (33.3)	23 (52.3)	0.059
**Pathogensis of stroke, (*****N*** **%)**				
Large artery atherosclerosis	41 (41.8)	21 (38.9)	20 (45.5)	0.512
Cardio-embolism	53 (54.1)	30 (55.6)	23 (52.3)	0.746
Anterior circulation stroke (*n* %)	90 (91.8)	49 (90.7)	41 (93.2)	0.661
Posterior circulation stroke (*n* %)	8 (8.2)	5 (9.3)	3 (6.8)	0.661
**Procedural options (*****N*** **%) and ICH (*****N*** **%) and recanalization**				
Aspiration	72 (73.5)	46 (85.2)	26 (59.1)	0.004 < 0.01
Combination of aspiration and stent retriever	17 (17.4)	5 (9.3)	12 (27.3)	0.019 < 0.05
Balloon angioplasty	8 (8.2)	3 (5.6)	5 (11.4)	0.296
Permanent stenting	5 (5.1)	2 (3.7)	3 (6.8)	0.486
Tirofiban administration	9 (9.2)	3 (5.6)	6 (13.6)	0.168
ICH	19 (19.4)	9 (16.7)	10 (22.7)	0.45
Recanalization	90 (91.8)	52 (96.3)	38 (86.4)	0.074

IQR, interquartile range; IVT, intravenous thrombolysis; NIHSS, National Institutes of Health Stroke Scale score; OTP time, onset to puncture time; OTR time, onset to recanalization time; ICH, intracranial hemorrhage.

### Efficacy results

**Figure 1:**
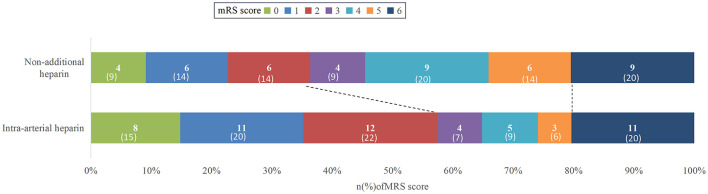
Clinical outcomes at 3 months by modified Rankin Scale (mRS) of patients with and without heparinization during mechanical thrombectomy.

Overall, 90 patients (91.8%) achieved successful arterial recanalization, with 52 patients (96.3%) in the arterial-heparin group and 38 patients (86.4%) in the non-additional heparin group. No significant inter-group difference was found in recanalization rates (*P* = 0.074). At 3 months follow-up, 47 patients (47.9%) achieved functional independence, and 20 patients (20.4%) died. The arterial-heparin group had a significantly higher rate of functional independence compared to the non-heparin group (57.4 vs. 36.4%, adjusted *P* = 0.035 < 0.05). There were no observed differences in mortality rates between the groups (adjusted *P* = 0.624; Table 2 and Figure 1).

**Table 2:** Safety and efficacy endpoints in patients grouped with or not heparin.

**Safety and efficacy endpoints**	**ALL (*n* = 98)**	**Intra-arterial Heparin (*n* = 54)**	**Non-additional heparin (*n* = 44)**	**OR (95%CI)**	***P*-value**	**Adjusted OR (95%CI)**	**Adjusted *P*-value**
**All patients (*****N*** = **98)**							
sICH	7 (7.1)	4 (7.4)	3 (6.8)	1.093 (0.231–5.166)	0.91	2.974 (0.341–25.955)	0.324
Distal embolization	5 (5.1)	1 (1.9)	4 (9.1)	0.189 (0.020–1.754)	0.143	0.155 (0.012–1.927)	0.147
Functional independence	47 (48)	31 (57.4)	16 (36.4)	2.359 (1.041–5.342)	0.038	3.282 (1.084–9.937)	0.035
Mortality	20 (20.4)	11 (20.4)	9 (20.5)	0.995 (0.371–2.671)	0.992	1.357 (0.400–4.598)	0.624

OR, odds ratio; sICH, symptomatic intracranial hemorrhage; Functional independence, mRS0–2. Adjusted for age, anticoagulation, Bridging IVT, OTP, OTR, smoking, aspiration, combination of aspiration and stent retriever, Tirofiban administration.

### Safety results

Overall, seven patients (7.1%) experienced sICH within 24 h post-MT, with four patients (7.4%) in the arterial-heparin group and three patients (6.8%) in the non-additional heparin group. Additionally, 19 patients (19.4%) experienced total intracerebral hemorrhage (ICH), and four patients (4.1%) had distal embolization. After adjusting for potential confounding factors, there were no significant differences in the incidence of sICH (*P* = 0.413) and distal embolization (adjusted *P* = 0.120) between the groups (Table 2).

### sICH and unfavorable prognosis

Multivariate logistic regression analysis suggested that direct aspiration may be a protective factor associated with sICH [odds ratio 0.009, (0.00–0.845), *P* = 0.042]. However, the admission NIHSS score [odds ratio 1.340, (1.144–1.570), *P* < 0.01] was identified as independent predictor of unfavorable outcomes, while post-operative anticoagulant therapy during hospitalization [odds ratio 9.591, (2.245–40.976), *P* = 0.02] was a protective factor (Table 3).

**Table 3:** Univariable and multivariable logistic regression analyses of the associations of intra-arterial heparin and other baseline characteristics with the probability of sICH and poor outcome following mechanical thrombectomy in patients with emergent large vessel occlusion.

**Variable**	**sICH**				**Poor outcome (mRS** ≥**3)**			
	**Univariable logistic**		**Multivariable logistic**		**Univariable logistic**		**Multivariable logistic**	
	**regression analysis**		**regression analysis**		**regression analysis**		**regression analysis**	
	**Odds ratio (95% CI)**	***P*** **-value**	**Odds ratio (95% CI)**	***P*** **-value**	**Odds ratio (95% CI)**	***P*** **-value**	**Odds ratio (95% CI)**	***P*** **-value**
Age	1.021 (0.969–1.076)	0.431	1.022 (0.945–1.106)	0.583	1.004 (0.976–1.034)	0.775	1.022 (0.969–1.077)	0.429
Bridging IVT	2.531 (0.291–22.042)	0.4	0.746 (0.048–11.595)	0.834	0.892 (0.371–2.144)	0.798	1.394 (0.360–5.392)	0.630
Admission NIHSS	0.932 (0.788–1.101)	0.408	00.853 (0.621–1.172)	0.327	1.225 (1.096–1.370)	< 0.01	1.340 (1.144–1.570)	< 0.01
Hypertension	0.767 (0.162–3.621)	0.737	1.705 (0.190–15.309)	0.634	0.608 (0.274–1.353)	0.223	0.526 (0.160–1.729)	0.290
Moderate to heavy alcohol	0.948 (0.173–5.192)	0.951	0.434 (0.036–5.238)	0.511	1.239 (0.520–2.953)	0.629	1.079 (0.210–5.556)	0.927
Cardio-embolism	0.615 (0.130–2.906)	0.539	1.470 (0.089–24.320)	0.788	0.792 (0.357–1.755)	0.565	0.785 (0.176–3.502)	0.751
Posterior circulation stroke	0.00 (0.00–inf)	0.999	0.00 (0.00–inf)	0.999	1.905 (0.429–8.452)	0397	1.750 (0.179–17.090)	0.63
Intra-arterial heparin	1.093 (0.231–5.166)	0.91	5.340 (0.353–80.7753)	0.227	2.359 (1.041–5.342)	0.040	3.076 (0.757–12.052)	0.116
Previous or current smoker	1.946 (0.411–9.208)	0.401	3.806 (0.287–50.529)	0.311	0.756 (0.337–1.692)	0.496	0.960 (0.192–4.789)	0.96
anticoagulation	0.00 (0.00–inf)	0.998	0.00 (0.00–inf)	0.997	3.200 (1.328–7.711)	0.010	9.591 (2.245–40.976)	0.002
Aspiration	0.451 (0.094–2.167)	0.32	0.009 (0.00–0.845)	0.042	2.111 (0.832–5.356)	0.116	1.384 (0.172–11.155)	0.76
Combination of aspiration and stent retriever	0.781 (0.088–6.944)	0.825	0.018 (0.00–2.063)	0.097	0.387 (0.125–1.199)	0.100	2.996 (0.261–34.418)	0.378
Distal embolization	0.00 (0.00–inf)	0.999	0.00 (0.00–inf)	0.999	0.255 (0.028–2.372)	0.230	1.581 (0.079–31.547)	0.164

sICH, symptomatic intracranial hemorrhage; CI, confidence interval; IVT, intravenous thrombolysis; NIHSS, National Institutes of Health Stroke Scale.

## Discussion

In this retrospective analysis, we found that continuous intra-arterial infusion of heparin administration during mechanical thrombectomy (MT) was associated with a significantly higher proportion of patients achieving long-term functional independence compared to the non-heparin group. However, there were no significant differences between the two groups regarding the incidence of sICH, mortality rates and distal embolization events, additionally, regression analysis indicates that baseline NIHSS score at admission is an independent risk factor for favorable prognosis, while anticoagulation therapy administered during hospitalization may act as a protective factor. Therefore, our results suggest that continuous intra-arterial infusion of heparin administration during MT for LVO-AIS may offer greater efficacy and relative safety compared to no additional administering heparin. But, given the observational design, these findings should be interpreted as exploratory and hypothesis-generating rather than practice-changing. Further randomized controlled trials are needed to confirm these observations.

The role of periprocedural heparin during MT remains controversial. While heparin has traditionally been used to prevent catheter-related thrombosis and distal embolic events (6), recent high-impact evidence has raised concerns regarding its routine use. A 2024 systematic review and meta-analysis reported that periprocedural heparin administration was not associated with improved functional outcomes and may increase the risk of distal embolization, with a potential signal of harm in patients receiving bridging intravenous thrombolysis (7). In addition, registry-based and multicenter observational studies published in 2024 suggested increased risks of sICH or unfavorable outcomes associated with intravenous unfractionated heparin bolus during MT (8, 9). A subgroup analysis of the MOST randomized clinical trial in *JAMA Neurology* found that the addition of intravenous argatroban or eptifibatide to standard care for patients undergoing mechanical thrombectomy did not improve reperfusion or clinical outcomes (10). These data have contributed to a growing consensus that indiscriminate systemic anticoagulant therapy during MT may be harmful, it is crucial to balance the potential benefits of heparinization against the potential dose-dependent risks of bleeding complications.

It is important to note, however, that most contemporary studies have focused on systemic intravenous bolus administration of heparin. In contrast, the strategy evaluated in the present study involved continuous intra-arterial heparin administration as part of the catheter flushing system, rather than systemic bolus dosing. Recent international surveys have demonstrated substantial heterogeneity in real-world intraprocedural anticoagulation practices, particularly regarding heparin concentrations in flushing solutions and routes of administration, underscoring the lack of standardized protocols (5). Differences in administration route, dose exposure, and pharmacokinetics may partly explain the heterogeneity of reported safety and efficacy outcomes across studies.

Earlier studies from the pre-modern thrombectomy era, including *post hoc* analyses of the Multi MERCI (3) and TREVO 2 (2) trials, suggested that heparin use during endovascular treatment was not associated with increased hemorrhagic complications and might be linked to improved functional outcomes. Reperfusion can increase sICH risk (11), and thus heparin may act on microcirculatory thrombosis and reduce the risk of sICH (12). However, these studies were conducted with earlier-generation devices and were characterized by imbalances in bridging intravenous thrombolysis and procedural techniques. More recent randomized and *post hoc* analyses, such as those from the MR CLEAN-MED trial, have demonstrated increased hemorrhagic risk without functional benefit from periprocedural intravenous aspirin or heparin, reinforcing the need for caution when interpreting earlier findings (13).

The PROACT trial, a randomized controlled study of intra-arterial recombinant urokinase, compared higher (100 IU/kg bolus followed by 1,000 IU/h) and lower (2,000 IU bolus followed by 500 IU/h) heparin dosing regimens (14). The results indicated that heparin has a dose-dependent effect on recanalization and sICH. A retrospective analysis by Chinese researchers on heparinization during MT found that excessive heparinization might be associated with increased risks of sICH and distal embolization, as well as worse long-term outcomes, highlighting the need for further randomized controlled trials (15). This suggests that appropriate dosing and administration methods for heparin during MT are crucial. In this retrospective study, the heparin administration method used during MT did not result in a statistically significant difference in sICH risk between the groups.

Several studies have investigated the use of intravenous heparin during MT (3, 4, 16, 17). The incidence of sICH reported in these studies ranges from 5 to 12%, which is consistent with our study's findings. However, in terms of efficacy endpoints, the risk of sICH seems to be outweighed by the higher rates of favorable outcomes, suggesting that selective use of heparin during MT may be beneficial.

Distal embolization results from thrombus fragmentation during the thrombectomy process and is associated with poorer clinical outcomes. In our non-heparin group, four cases (9.1%) of distal embolization were recorded, which although not statistically different from the heparin group (1.9%, *P* = 0.143), was higher than previously reported rates (18). This may suggests that the appropriate use of heparin via high-pressure infusion may be more effective in preventing distal embolization. In addition, our multivariate regression analysis indicated that the use of direct aspiration may serve as a protective factor against sICH, we believe this could be attributed to the reduced endothelial irritation of the target vessel associated with direct aspiration. It is noteworthy that several infinite values were observed, and multiple 95% confidence intervals were excessively wide, which may be attributable to the limited sample size and event rates of our study.

In terms of baseline characteristics, the heparin group had a higher proportion of patients who received bridging therapy [43 (79.6%) vs. 27 (61.4%)], post-operative anticoagulation [24 (44.4%) vs. 9 (20.5%)] and direct aspiration thrombectomy [46 (85.2%) vs. 26 (59.1%)]. In contrast, the non-heparin group had a higher proportion treated with combined aspiration and stent retriever techniques [12 (27.3%) vs. 5 (9.3%)]. Although multivariable regression was performed, residual confounding cannot be excluded. Differences in patient presentation, stroke etiology, and operator preference may also have influenced treatment decisions and outcomes.

In addition, a higher baseline NIHSS score at admission was found to be independently associated with poor functional outcomes, which may reflect the established relationship between the severity of initial neurological deficits and clinical prognosis.

Interestingly, anticoagulation therapy initiated during hospitalization appeared to be associated with improved outcomes. This observation may be partially supported by recent studies advocating for early anticoagulation strategies following acute ischemic stroke (19, 20). This association, although not conclusive, may suggest a potential direction for future prospective investigation.

Overall, this study proposes the hypothesis that continuous intra-arterial infusion of heparin administration during MT may represent a feasible and relatively safe periprocedural strategy in selected patients. However, given the retrospective design, single-center setting, baseline imbalances, and limited sample size, the findings should be interpreted with caution. Prospective, standardized, and ideally randomized studies are required before any modification of clinical practice can be recommended.

### Limitations

The main advantage of this study is the introduction of a novel heparin administration protocol during MT, which has been preliminarily validated for both efficacy and safety. However, there are several limitations: (1) retrospective nature: being a retrospective analysis, this study cannot establish causality. (2) sample size and generalizability: the sample size is small and limited to patients from a single center, which may introduce bias in statistical results and limit the generalizability of the findings to a broader population. (3) Lack of dose stratification: the study did not stratify heparin dosing based on patient's weight and etiology. (4) Absence of monitoring: there was no monitoring of activated clotting time (ACT) to assess the degree of heparinization. (5) Absence of baseline ASPECTS scores: baseline ASPECTS scores were not recorded, preventing us from excluding the possibility that the control group included more patients with large core infarcts, which may have affected the rate of favorable outcomes. (6) Outcome adjudication was not fully blinded, which may introduce potential assessment bias.

Therefore, further prospective randomized controlled trials are needed to validate these conclusions.

## Conclusion

Our study suggests that, in real-world practice, continuous intra-arterial infusion of heparin administration during MT for acute large vessel occlusion ischemic stroke may be safe and is associated with higher rates of favorable outcomes. Further prospective research is needed to validate these findings.

## Data Availability

The raw data supporting the conclusions of this article will be made available by the authors, without undue reservation.
